# When, Who, and How to Sample: Designing Practical Surveillance for 7 Neglected Tropical Diseases as We Approach Elimination

**DOI:** 10.1093/infdis/jiaa198

**Published:** 2020-06-11

**Authors:** Jaspreet Toor, Luc E Coffeng, Jonathan I D Hamley, Claudio Fronterre, Joaquin M Prada, M Soledad Castaño, Emma L Davis, William Godwin, Andreia Vasconcelos, Graham F Medley, T Déirdre Hollingsworth

**Affiliations:** 1 Big Data Institute, Li Ka Shing Centre for Health Information and Discovery, University of Oxford, Oxford, United Kingdom; 2 Department of Public Health, Erasmus Medical Center, University Medical Center Rotterdam, Rotterdam, The Netherlands; 3 London Centre for Neglected Tropical Disease Research, Department of Infectious Disease Epidemiology, Imperial College London, London, United Kingdom; 4 Medical Research Council Centre for Global Infectious Disease Analysis, Department of Infectious Disease Epidemiology, Imperial College London, London, United Kingdom; 5 Centre for Health Informatics, Computing, and Statistics, Lancaster University, Lancaster, United Kingdom; 6 School of Veterinary Medicine, Faculty of Health and Medical Sciences, University of Surrey, Guildford, United Kingdom; 7 Department of Epidemiology and Public Health, Swiss Tropical and Public Health Institute, University of Basel, Basel, Switzerland; 8 Francis I. Proctor Foundation, University of California San Francisco, San Francisco, California, USA; 9 Centre for Mathematical Modelling of Infectious Disease, London School of Hygiene and Tropical Medicine, London, United Kingdom

**Keywords:** surveillance, elimination modeling, prevalence threshold

## Abstract

As neglected tropical disease programs look to consolidate the successes of moving towards elimination, we need to understand the dynamics of transmission at low prevalence to inform surveillance strategies for detecting elimination and resurgence. In this special collection, modelling insights are used to highlight drivers of local elimination, evaluate strategies for detecting resurgence, and show the importance of rational spatial sampling schemes for several neglected tropical diseases (specifically schistosomiasis, soil-transmitted helminths, lymphatic filariasis, trachoma, onchocerciasis, visceral leishmaniasis, and gambiense sleeping sickness).

## WHERE WE HAVE BEEN...

Mathematical modelling of neglected tropical diseases (NTDs) has strengthened and diversified over the past decade, in parallel with extraordinary achievements in scaling up NTD programs. The NTD Modelling Consortium, a research project funded by the Bill and Melinda Gates Foundation, was formed in 2014 to support the efforts around a small number of key diseases [1]. The consortium was formed of at least 2 international modelling groups working on each disease to provide robust scientific insight, based on the available data. One of the priority tasks was to evaluate strategies to achieve the World Health Organization 2020 goals [2]. Whilst providing evidence-based modelling recommendations to inform policy makers, the process of model comparison is critical to the development of models, improving the robustness of results and thereby supporting policy makers to trust the models [3, 4]. However, this is not a static process as model developments and comparisons have continued to respond to an evolving evidence base [5]. The consortium has also identified data needs across surveillance programs to monitor progress and to address epidemiological uncertainties to strengthen the scientific evidence underlying the model assumptions and inform programs more directly [6].

As NTD programs move towards achieving their goals and deciding whether they can stop treatment, there will be a critical shift towards potential elimination, with the accompanying demand for more robust surveillance in order to ensure that the hard-won gains are not lost. In this special collection, we investigate the dynamics of low-prevalence transmission for several NTDs, namely, schistosomiasis, soil-transmitted helminths, lymphatic filariasis, trachoma, onchocerciasis, visceral leishmaniasis, and gambiense sleeping sickness. We provide guidance on the surveillance that will be required once programs are halted.

## ROLLING THE DICE: DYNAMICS OF ELIMINATION AND RESURGENCE

**Figure 1.**
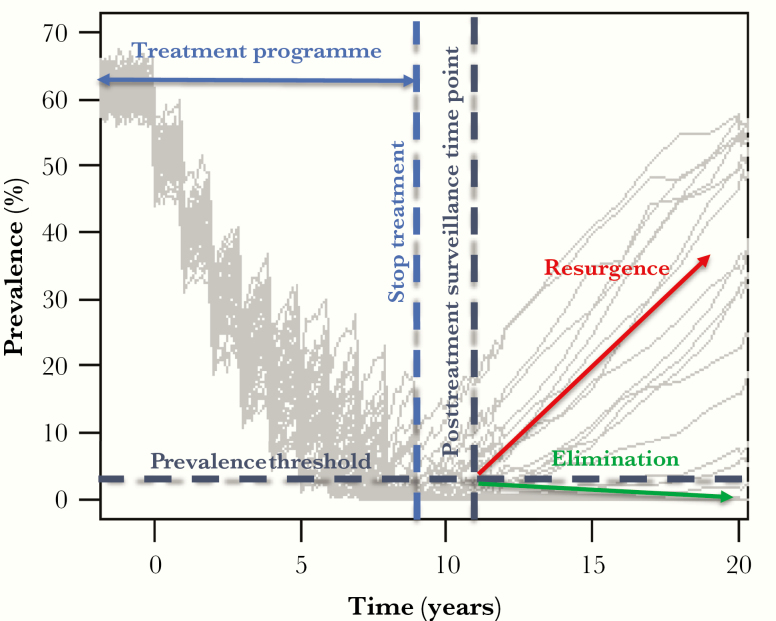
Elimination dynamics: simulations showing the stochasticity around achieving elimination or resurgence after stopping a mass treatment program (figure adapted from [7] for schistosomiasis; http://creativecommons.org/licenses/by/4.0/). At the posttreatment surveillance time point, if the prevalence threshold is reached, we can predict with a certain probability that elimination will occur (likewise, if we are above this threshold, we can predict that resurgence will occur). Multiple surveillance time points will likely be required at a frequency depending on the disease because resurgence will always be a risk before diseases are eradicated.

The NTDs are unusual in that interventions, particularly mass drug administration (MDA), are often halted, unlike vaccine-preventable diseases which typically have longer-lasting programs. Once interventions reduce the prevalence of an infection to a low level, the final few cases may infect few or no other cases and the infection may be eliminated. There is stochasticity surrounding this process, with chance events leading to either elimination or resurgence (Figure 1). However, there are conditions that can weigh the probability of elimination and drive it higher or lower.

It is important to remember that for many NTDs reinfection can occur, and this also means that in theory prevalence could return to preintervention levels once programs are halted. Slow epidemic growth rates for these infections means that there are opportunities to detect resurgence but, equally, as resurgence could take years to detect, the political will may have waned by the time they are detected, and screening programs will have to be in place throughout this time to detect these resurgent cases.

The most important driver of resurgence is the underlying transmission rate. For the NTDs addressed in this collection, the first line of attack is targeted at treatment of human cases, to reduce the burden of disease. However, as case numbers drop and elimination comes into prospect, transmission reduction through other interventions, such as vector control and sanitation, becomes crucial in reducing the probability and speed of resurgence, particularly when MDA or screening programs are halted. Surveillance activities for detecting elimination and resurgence become increasingly important to ensure that successes are maintained.

## BALANCING ACT: DESIGNING PRACTICAL SURVEILLANCE SCHEMES FOR ELIMINATION

When targeting elimination of NTDs across large areas, it is impossible to sample everyone or every community to confirm that the incidence of infection has been reduced to zero. Therefore any surveillance scheme is inherently associated with some degree of uncertainty. The greater the “effort” made in surveillance, the lower this uncertainty. Such surveillance means an increase in costs. However, once elimination is affirmed, the costs stop whilst the benefits continue to accumulate over time [8]. Hence, bigger efforts for detecting elimination may be more cost-effective, or even cost-saving, in the long term.

Given the difficulty in measuring zero incidence, practical thresholds can be determined and used in lieu of this. These thresholds are typically based on a prevalence of infection measure, whereby once this prevalence is reached, elimination or resurgence probabilities can be estimated. It is important to remember that whatever the threshold, there is some probability that resurgence will happen, and postvalidation surveillance is essential. The level of accuracy required, or imposed by cost constraints, will affect the surveillance criteria, that is specifying a 90% certainty of elimination will mean we can have a higher threshold for stopping treatment relative to 95% certainty. For schistosomiasis, by reaching 1% Kato-Katz prevalence 2 years after stopping MDA we can be 90% certain elimination will occur, whereas the certainty is 75% once 5% prevalence has been reached [7]. In addition to this threshold, surveillance schemes also need to provide guidance on when to sample, who to sample, and how to sample.

## KEY CONSIDERATIONS

### When to Sample

Most NTDs have slow epidemic growth rates and, therefore, if there is resurgence, it may take years to be detected. Likewise, elimination can take years to occur as low-prevalence settings may approach zero slowly. Surveillance to detect elimination or resurgence will usually need to be carried out more than once depending on the disease. For lymphatic filariasis, resurgence can occur outside of the currently recommended surveillance period of 4–6 years. Hence, post-MDA monitoring would be needed for longer to ensure success [9].

**Figure 2.**
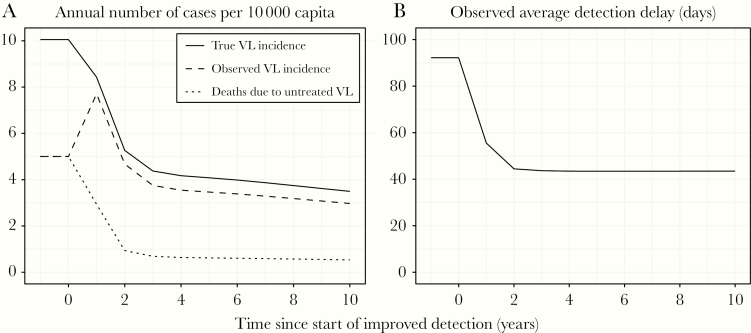
Model predictions for visceral leishmaniasis (VL) showing the impact of improved detection vs prior to improved detection where 50% of cases died before detection (figure from [10]; http://creativecommons.org/licenses/by/4.0/).

For diseases where case management is the main strategy, intensified testing and case finding is important (Figure 2). The timing of stepping back active and passive case detection is key. Frequently, the treatment of cases is the principal intervention, that is surveillance and control are combined. Active surveillance showing no detected cases over multiple years can provide more confidence of elimination, relative to a single year of active surveillance as shown for gambiense sleeping sickness [11]. As cases fall, there are risks of declining quality of surveillance, including higher delays in case detection times and losses in skills of identifying cases. Hence, monitoring the performance of case detection programs is important and incidence alone may not be a sufficient measure. For visceral leishmaniasis, although relaxation of detection efforts may lead to an initial decline in observed incidence, this comes with a high risk of resurgence. Here, the duration of symptoms in detected cases can be informative in assessing the performance of case detection programs [10]. For such diseases, finding an alternative means of assessing transmission is an invaluable surveillance activity to ensure elimination [12].

### Who to Sample

The criteria of who to sample can vary from sampling a specific number of people from the whole population at random to sampling more purposively from a particular subgroup(s). This decision depends on the epidemiology of the disease, particularly the age-dependent exposure rates. These rates can vary geographically across communities and countries. Data required to inform this (ie, infection data across all age groups) are limited for the NTDs. Hence, we have to use assumptions regarding epidemiology but these will affect the efficacy of the threshold. For onchocerciasis, assuming different exposure patterns results in either children younger than 10 years old or children 5–14 years old being the most informative age groups for serological monitoring [5]. The analyses in this collection have been careful to examine the uncertainty surrounding these conclusions and identify focused empirical studies that would inform these estimates and improve the robustness of such results.

Notably, the decision of who to sample also needs to be made at the early stages of a program as this plays a role in determining the optimal treatment strategy. For schistosomiasis, children and adults need to be sampled, particularly in high-prevalence settings, in order to determine the coverage levels required within a program [13].

### How to Sample

The question of how to sample revolves around the diagnostic technique that has been used to measure the threshold for detecting elimination. As diagnostic tests for NTDs differ in terms of their sensitivity and specificity, the diagnostic used will impact the surveillance criteria and the certainty of our measures. Better diagnostics will allow policy makers to make the most cost-effective decisions, particularly as they will improve the detection of elimination and resurgence [14]. This is evident with trachoma, where an increase in trachomatous inflammation-follicular prevalence in children 1–9 years old may be a result of resurgence or measurement error [15].

The budget for surveillance will play a role given that the costs associated with each diagnostic approach will vary. Despite new techniques having potentially higher unit costs, this may be outweighed by the long-term programmatic benefits of being able to detect elimination [14]. For the NTDs such as the soil-transmitted helminths and schistosomiasis, given limited resources, multiple samples per individual means sampling fewer individuals. Using a set budget for the soil-transmitted helminths, a single-slide Kato-Katz technique would be sufficient to evaluate both prevalence of any and moderate-to-heavy intensity of infection during the early program stages, whereas more sensitive sampling schemes may be required for decisions involving stopping MDA [16].

### Spatial Heterogeneity

When designing surveillance schemes, spatial heterogeneity cannot be ignored. This is not a surprise, but it is surprising how little consideration there has been on how space and time interact in elimination dynamics. Fronterre et al [17] demonstrate the impact of spatial heterogeneity on the design and analysis of surveys aimed at assessing elimination, with a focus on lymphatic filariasis. They demonstrate the enormous improvement in statistical efficiency that can be obtained by taking proper account of spatial correlations (ie, that places nearer to each other are more similar) when estimating prevalence. Whilst these expected gains in efficiency are disease and country specific, the general methodology can be applied to all infectious diseases.

## WHERE WE ARE GOING...

With elimination as the end goal for NTD programs, it is not feasible to sample everyone everywhere once programs are halted, so practical surveillance schemes are needed. Such surveillance is of great importance to ensure that the hard-won gains are maintained. In this special collection, we use modelling insights to provide highly valuable guidance on the surveillance that is required. To improve and validate the guidance, models are demanding more data. Additionally, better diagnostics will improve the quality of the data and the decisions made. Looking ahead to a hopeful future with well-designed surveillance schemes, the certainty with which decision makers can certify elimination will beneficially improve, allowing us to rightfully celebrate reaching this end goal for NTDs.

The papers in this collection were written during 2019, before coronavirus 2019 (COVID-19) emerged as a pandemic. This will likely be the biggest global public health and economic crisis for decades. Its impact on NTD elimination is still to be determined. On one hand the cessation of interventions is likely to see local resurgence for some diseases, but longer-term public health and the necessity for surveillance may have a higher profile. Understanding the situation after the pandemic has subsided will be a major undertaking—well-designed, cost-effective surveillance is always critically important.

## Notes

***Financial support.*** This work was supported by the Bill and Melinda Gates Foundation through the Neglected Tropical Disease Modelling Consortium (grant number OPP1184344); and the Dutch Research Council (grant number 016.Veni.178.023 to L. E. C.).

***Supplement sponsorship.*** The authors acknowledge funding of the NTD Modelling Consortium by the Bill and Melinda Gates Foundation (OPP1184344).

***Potential conflicts of interest.*** All authors: No reported conflicts of interest. All authors have submitted the ICMJE Form for Disclosure of Potential Conflicts of Interest. Conflicts that the editors consider relevant to the content of the manuscript have been disclosed.
